# Per- and Polyfluoroalkyl Substances in Early Pregnancy and Risk for Preeclampsia: A Case-Control Study in Southern Sweden

**DOI:** 10.3390/toxics8020043

**Published:** 2020-06-16

**Authors:** Lars Rylander, Christian H. Lindh, Stefan R. Hansson, Karin Broberg, Karin Källén

**Affiliations:** 1 Division of Occupational and Environmental Medicine, Department of Laboratory Medicine, Lund University, MV (Building 402a), 223 81 Lund, Sweden; christian.lindh@med.lu.se (C.H.L.); karin.broberg@med.lu.se (K.B.); karin.kallen@med.lu.se (K.K.); 2 Division of Obstetrics and Gynecology, Department of Clinical Sciences Lund, Lund University, 221 85 Lund, Sweden; stefan.hansson@med.lu.se; 3 Institute of Environmental Medicine, Karolinska Institute, Nobels väg 13, 171 65 Solna, Sweden; 4 Tornblad Institute, Lund University, Biskopsgatan 7, 223 62 Lund, Sweden

**Keywords:** PFAS, PFOA, PFOS, PFNA, PFHxS, preeclampsia, biobank, register, Sweden

## Abstract

Preeclampsia is one of the most common causes of perinatal and maternal morbidity/mortality. One suggested environmental risk factor is exposure to endocrine-disrupting pollutants such as per- and polyfluoroalkyl substances (PFAS). The present case-control study in southern Sweden aims to investigate the hypothesized association between serum concentrations of PFAS in early pregnancy and the risk of developing preeclampsia. The study included 296 women diagnosed with preeclampsia (cases) and 580 healthy pregnant women (controls). Maternal serum samples were obtained from a biobank of samples collected in early pregnancy in connection with screening for infections. Serum concentrations of perfluorooctanoic acid (PFOA), perfluorooctane sulfonate (PFOS), perfluorononanoic acid (PFNA), and perfluorohexane sulfonate (PFHxS) were analyzed using liquid chromatography-tandem-mass-spectrometry (LC/MS/MS). Among primiparous women, there were no differences in PFAS concentrations in early pregnancy between the cases and the controls whereas among multipara women, the cases had significantly higher concentrations of PFNA (median concentrations were 0.44 and 0.38 ng/mL, *p* = 0.04). When individual PFAS were categorized into quartiles and adjustment for potential confounders was performed, the women in the highest quartiles had no significant increased risks of developing preeclampsia as compared with women in the lowest category. In conclusion, the present study provides limited support for the hypothesized association between PFAS and preeclampsia in a population with relatively low exposure levels.

## 1. Introduction

Preeclampsia affects 3–7 percent of all pregnancies each year and is one of the most common causes of perinatal and maternal morbidity and mortality [1,2]. It has been estimated that 8.5 million pregnant women worldwide are affected annually. Preeclampsia has been called "the disease of theories" because so much is unknown, and many different explanations for the disease have been proposed over the years [3]. There are some factors which increase the risk for preeclampsia, such as being primipara, having a history of preeclampsia, high maternal body mass index (BMI), presence of certain systemic diseases such as diabetes and hypertension, African ethnicity, increased maternal age, and long inter-pregnancy interval, whereas smoking has been observed to decrease the risk [3]. However, overall, these factors only explain a relatively small portion of the preeclampsia cases and any "new" risk factor that can be identified and eliminated/reduced is of great importance. One group of environmental factors that has recently been suggested as a risk factor is exposure to endocrine-disrupting chemicals (EDCs) [4,5].

EDCs are chemicals that are present in our environment which affect and/or interact with our normal hormonal systems [6]. They are large group of chemicals (such as chlorinated, brominated and fluorinated substances, phthalates and bisphenol A) with many different properties and areas of use. Some of these chemicals are very persistent. An example of this is the per- and polyfluoroalkyl substances (PFAS) with half-lives of several years [7,8]. PFAS have been produced since the 1950s and used as industrial surfactants, in water, stain, and grease-resistant coatings in consumer goods (e.g., non-stick pans, raincoats, fire-fighting foams) [9]. Human exposure is primarily via diet, but also via drinking water due to widespread surface and groundwater contamination [10]. The mechanism behind the suggested association between PFAS and preeclampsia is unknown, but it is reasonable to believe that vascular disturbances are significant. One of the critical effects of PFAS exposure identified in a recent opinion by the European Food Safety Authority (EFSA) was an association with cholesterol levels, which might be seen as indirect support for the suspicion that vascular disturbances are involved in the mechanism [11]. One can also speculate on pathways where exposure to PFAS with its endocrine-disrupting properties could potentially lead to oxidative stress and inflammation.

Two epidemiological studies from the US, which were part of the C8 Health Project, showed statistically significant associations between exposure to two PFAS, perfluorooctanoic acid (PFOA) and perfluorooctane sulfonate (PFOS), and preeclampsia [12,13,14]. A weakness of these studies was the use of indirect methods to assess PFAS during pregnancy, either by the use of predictive modelling to estimate the exposure at the time of pregnancy [12] or by measuring the exposure up to five years after pregnancy [13]. A study within the Norwegian Mother, Father and Child Cohort (MoBa), in which PFAS were measured at mid-pregnancy, i.e., before the onset of preeclampsia, did not show any statistically significant associations between PFAS and preeclampsia [15]. Based on these studies, the EFSA opinion stated that there is still insufficient evidence to suggest that PFOS and PFOA, the only PFAS evaluated in the opinion, are associated with preeclampsia [11]. After the opinion was published, one study from Sweden showed statistically significant associations between PFOS and perfluorononanoic acid (PFNA) and preeclampsia [16], and one study from China showed significant associations with perfluorobutane sulfonate (PFBS), perfluorohexane sulfonate (PFHxS), and perfluoroundecanoic acid (PFUA) [17]. However, these studies included relatively few cases. Therefore, there is a great need for population-based studies with high participation rates as well as valid data regarding outcome, exposure, and potential confounders in order to further clarify whether there is a causal link between PFAS and preeclampsia. Accordingly, the present study aimed to investigate the hypothesized association between PFAS in early pregnancy and the risk for developing preeclampsia by combining comprehensive information from registers and analyzing the concentrations of PFAS in maternal serum collected in early pregnancy before the manifestation of preeclampsia.

## 2. Material and Methods

### 2.1. Study Design and Sources

The study, which was approved by the Ethical committee (Reg. No. 2014/696; approval date 30 October 2014) at Lund University, Sweden, was conducted as a case-control study where the following sources were used:

#### 2.1.1. Biobanked Serum Samples

The Southern Sweden Maternal Biobank (SSMB) is a biobank in Scania (in Swedish: Skåne), the most Southern county in Sweden, which started in 1989 and contains >250,000 maternal serum samples collected in early pregnancy (around 12–14 weeks). The serum samples were taken in conjunction with screening for infections and German measles (rubella) [18].

#### 2.1.2. Register Data

The Swedish Medical Birth Registry (MBR) includes virtually all children born in Sweden from 1973 onwards [19]. The register was used in combination with a local birth register for Scania, the Perinatal Revision South (PRS) [20]. In addition, ultrasound visits in early and late pregnancy for virtually all women have been performed in parts of Scania and registered in a separate database. 

The study size was based on an a priori power calculation. With the assumption that 20% of the controls were defined as exposed and, based on 300 included preeclampsia cases and 600 controls with 80% statistical power, it was possible to detect an odds ratio of 1.6 or higher with a statistical significance (*p* < 0.05). The assumption that 20% of the controls were exposed is arbitrary, and its biological relevance in different populations can be questioned. However, if we instead assumed a rate of 15% or 25%, the detectable odds ratio only changed marginally.

All cases and controls were identified from the birth register and the ultrasound database. Conditions and diagnoses were recorded using checkboxes and/or the International Classification of Diseases code (ICD), where the 9th revision was used before 1998 and the 10th revision from 1998 onwards. From the MBR, preeclampsia was identified through marked check boxes for moderate or severe preeclampsia or by the presence of ICD-codes 642E, 642F (ICD9), or O140, O141, or O149 (ICD10). The controls were randomly chosen among women who were not diagnosed with preeclampsia and whose children were not small for gestational age (SGA). The SGA diagnosis was defined as birth weight more than two standard deviations below the expected birth weight for gestational age and gender according to the Swedish intrauterine growth curve [21]. The reason women with an SGA child were excluded was due to the fact that the controls were included in an on-going study investigating the association between PFAS and SGA. 

A list of 450 randomly selected preeclampsia cases and 900 randomly selected controls was made and linked to the biobank. Four hundred and twenty out of the 450 (93%) cases were present in the biobank. The corresponding figure among the controls was 95% (*n* = 854). The list of preeclampsia cases and controls was randomly sorted, and the first 304 preeclampsia cases and 603 controls with serum samples in the biobank were selected. The reason that all 1350 samples were not analysed was to avoid exceeding the project budget and still have the possibility to detect significant associations of interest, which was based on the a priori power calculation. In eight preeclampsia cases and 23 controls, it was not possible to analyse PFAS concentrations due to the sample volume that was too small. The final number of participants were therefore 296 preeclampsia cases and 580 controls.

### 2.2. Analysis of PFAS

Serum concentrations of perfluorohexane sulfonate (PFHxS), perfluorooctanoic acid (PFOA), perfluorononanoic acid (PFNA), and perfluorooctane sulfonate (PFOS) were analysed in the laboratory of Occupational and Environmental Medicine at Lund University in Sweden using liquid chromatography-tandem-mass-spectrometry (LC/MS/MS). The samples were analysed according to a modified method by Lindh and colleagues [22]. In brief, aliquots of serum were added with labeled internal standards for all compounds. The proteins were precipitated with acetonitrile and vigorously shaken for 30 min. The samples were then centrifuged and analysed using a LC/MS/MS (QTRAP 5500, AB Sciex, Foster City, CA, USA). In each analytical batch, calibration standards, two homemade quality control (QC) samples and chemical blank samples were included. The samples were analysed in duplicate and in a randomized order. The limits of detection were 0.03 ng/mL for PFHxS and PFNA, 0.04 ng/mL for PFOA, and 0.12 ng/mL for PFOS. The coefficient of variation (CV) of the QC samples (*n* = 32) was 8% for PFHxS at 2 ng/mL and 10% at 3 ng/mL, for PFOA, 12% at 3 ng/mL and 9% at 4 ng/mL, for PFNA 10% at 2 ng/mL and 9% at 4 ng/mL, and for PFOS 7 % at 12 ng/mL and 8% at 13 ng/mL. The analyses of PFOS and PFOA are part of a quality control program between analytical laboratories coordinated by Professor Hans Drexler of the Institute and Outpatient Clinic for Occupational, Social and Environmental Medicine at the University of Erlangen-Nuremberg, Germany. The laboratory also participates in the ICI/EQUAS exercises for the analysis of PFHxS, PFOA, PFNA, and PFOS and is approved by the HBM4EU project.

### 2.3. Other Pregnancy Information

From the Medical Birth Registers, the following additional information was collected in early pregnancy; maternal body mass index (kg/m^2^), parity, maternal smoking (three categories: non-smokers, 1–9 cigarettes/day, and >9 cigarettes/day), diabetes (yes/no), gestational diabetes (yes/no), involuntary childlessness for at least one year (yes/no), and maternal country of origin (8 different categories, see Table 1).

### 2.4. Statistics

For pairwise correlations between the different PFAS, Spearman’s correlations coefficients was calculated. Separate analyses were performed for the preeclampsia cases and the controls. When the PFAS concentrations were used as continuous variables, we compared the concentrations between the primiparous and the multiparous women as well as between the cases and the controls with Mann-Whitney *U* tests. In addition, the concentrations of the different PFAS were categorized into quartiles based on the distributions among the controls. The associations between the categorized exposures and preeclampsia were evaluated using logistic regression models generating odds ratios (OR) and 95% confidence intervals (CI). The lowest exposure quartile was used as the reference category. In a first step we performed crude analyses, and in a second step, we performed a priori defined models where we adjusted for maternal age (4 categories: –25, 26–<30, 31–<35, and >35 years), BMI (4 categories: <20, 20–<25, 25–<30, ≥30 kg/m^2^), smoking (2 categories: yes and no), and parity (2 categories: 1 and ≥2). The other background variables in the present study were either not statistically significantly associated with preeclampsia or had categories with too few individuals. In addition, based on the knowledge that primiparous women have a pronounced increased risk for preeclampsia and the fact that some other studies only included primiparous women, we performed separate analyses for primiparous and multiparous women. Statistical significance was defined as *p*-values below 0.05 or 95% CIs not including 1.00. All analyses were performed with SPSS.

## 3. Results

There was a significantly higher fraction of primiparous women (77.0% vs. 47.1%; OR 3.77, 95% CI 2.75–5.17) and obese women among the cases as compared to the controls (BMI > 30; 17.7% vs. 9.5%; OR 1.99, 95% CI 1.26–3.14) (Table 1). In addition, the cases did significantly more often have diabetes (2.0% vs. 0.3%; OR 5.98, 95% CI 1.20–29.8), and their infants were more often born preterm (<37 weeks; 34.5% vs. 2.5%; OR 19.8, 95% CI 11.2–34.9).

With one exception (between PFOS and PFNA among the cases), pairwise correlations showed that the different PFAS were significantly correlated in the cases as well as among the controls (Table 2). The significant correlation coefficients varied from 0.14 (i.e., rather weak) to 0.68, with the strongest correlation observed between PFOA and PFOS.

The PFAS concentrations are shown in Table 3. The highest concentrations were observed for PFOS followed by PFOA. Among the controls, all individual PFAS concentrations were significantly higher among primiparous women as compared with the multiparous women (all *p*-values < 0.001), while between the cases, there were significant differences only for PFOA (*p* < 0.001) and PFHxS (*p* = 0.01). When comparing primipara cases with primipara controls, no significant differences were observed for the individual PFAS (all *p*-values ≥ 0.20), whereas for the corresponding comparisons among multipara cases and controls, the *p*-value was 0.04 for PFNA and 0.06 for PFHxS. 

When the PFAS were categorized into quartiles, the women in the two highest quartiles of PFOA (ORs 1.82 and 1.96), PFNA (ORs 1.83 and 1.83), and PFHxS (ORs 2.03 and 2.18) in the unadjusted models had a significantly increased risk of developing preeclampsia as compared to women in the lowest category (Table 4). Adjusting for potential confounders did decrease all estimates and, with one exception (3rd vs. 1st quartile for PFHxS), they were non-significant. Restricting the analyses to either primiparous or multiparous women, again with one exception (3rd vs. 1st quartile for PFHxS among multiparous women; OR 2.39, 95% CI 1.05–5.45) generated non-significant estimates in the adjusted analyses.

## 4. Discussion

In the unadjusted analyses, the present study showed statistically significant increased risks for developing preeclampsia among women from the general population in the southern part of Sweden with the highest concentrations of PFOA and PFHxS in early pregnancy. However, these associations were strongly confounded by parity and in the adjusted analyses, all ORs were clearly lowered and with one exception that was statistically non-significant. In addition, when PFAS concentrations were analysed as continuous variables, there were no significant differences in PFAS concentrations among primiparous women between the cases and the controls.

In the opinion published in 2018 by the European Food Safety Authority (EFSA), the identified critical effect was not preeclampsia but cholesterol levels in adults, antibody response at vaccination in children, reduced birth weight, and levels of the liver enzyme alanine aminotransferase (ALT) [11]. Regarding preeclampsia, the opinion stressed some weaknesses in the studies from the US where associations were found and concluded that there is insufficient evidence to suggest that PFOS or PFOA are associated with preeclampsia. 

The present study as well as the Norwegian study (within in the MOBA cohort) and a recently published study from Sweden (within the SELMA cohort) measured PFAS concentrations during pregnancy before preeclampsia was diagnosed [15,16]. The Norwegian study did not show associations between PFAS concentrations and preeclampsia, and the distributions of the PFAS concentrations were very similar to the findings in the present study [15]. The median concentration of PFOA among primipara controls in the present study was 3.0 ng/mL compared to 2.8 ng/mL in the Norwegian study. The corresponding figures for PFOS were 14.7 vs. 12.9 ng/mL, for PFNA 0.54 vs. 0.54 ng/mL, and for PFHxS 0.71 vs. 0.69 ng/mL. There are, however, some differences between the two studies: whereas the present study measured the PFAS concentrations in serum collected in early pregnancy, the Norwegian study measured the concentrations in plasma collected in mid-pregnancy. A second difference is the participation rate. The Norwegian study did have a participation rate below 40% compared to >90% of the selected individuals in the present study having serum samples in the biobank. A third difference was the possibility of adjusting for plasma creatinine or plasma cystatin C in the Norwegian study, which was not possible in the present study. On the other hand, the adjustment did not affect the estimates in the Norwegian study. A fourth difference between the studies was that the Norwegian study was restricted to nulliparous women whereas the present study also included multiparous women. Despite these differences, the overall conclusion from the two studies was very similar. However, the other study from Sweden within the SELMA cohort did find statistically significant associations between serum concentrations of PFOS and PFNA collected in early pregnancy (around gestational week 10) and preeclampsia [16]. The concentrations of PFNA in that study (median 0.53 ng/mL) and the present study were very similar, though we did not find statistically significant associations. Also, in contrast to Wikström and colleagues, we did not observe statistically significant associations between PFOS and preeclampsia, even though the median concentrations of PFOS were more than twice as high in the present study cohort (14.7 vs. 5.34 ng/mL). The reason for this discrepancy is unknown. It is of interest to note that the number of cases differed between the studies: 296 cases in the present study vs. 64 in the Wikström study.

The present study does have a number of strengths. The registers we used include in principle the entire population, and among the selected cases and controls, more than 90% had serum samples available in the biobank [19]. This means that the risk for selection bias is minor. In addition, the serum samples were collected at approximately the same number of gestational weeks in early pregnancy, before the onset of preeclampsia. Moreover, the preeclampsia diagnosis was based on register information and not on self-reported data. The PFAS were analysed with validated methods in a certified and recognized laboratory [23]. A weakness of the present study is the lack of information regarding heredity, but it is unlikely that heredity is associated with PFAS levels in early pregnancy. Moreover, the number of preterm births among the controls was too small to draw any firm conclusions on the importance of this variable.

## 5. Conclusions

In conclusion, the present study, which was performed on the general population with relatively low exposure concentrations of PFAS provided limited support for the hypothesized association between maternal serum concentrations of PFAS in early pregnancy and preeclampsia. The associations observed in the unadjusted analyses were strongly confounded by parity. The conclusions from the present study coincide with the conclusions of a large study from Norway, while another recent study did show significant associations. The reasons for these discrepancies have to be further investigated as well as the importance of PFAS in populations with much higher exposures.

## Figures and Tables

**Table 1:** Background characteristics among 296 preeclampsia cases and 580 controls. Odds ratios (OR) and 95% confidence intervals (CI) were obtained from univariate logistic regression models.

Characteristics	Mean/Median (min, max)		OR	95% CI
	Cases	Controls		
Maternal birth year	1973/1974 (1952, 1992)	1973/1973 (1955, 1993)	- ^a^	-
Calendar-year of childbirth	2004/2005 (1995, 2009)	2003/2004 (1995, 2009)	-	-
Maternal age at childbirth (year)	30/30 (15, 44)	29/29 (15, 42)	-	-
**Characteristics**	***n* (%)**		**OR**	**95% CI**
	**Cases**	**Controls**		
BMI in early pregnancy (kg/m^2^)				
<20	21 (8.4)	60 (11.9)	0.76	0.44–1.30
20–<25	132 (53.0)	286 (56.9)	1.00	Ref
25–<30	52 (20.9)	109 (21.7)	1.03	0.70–1.53
≥30	44 (17.7)	48 (9.5)	1.99	1.26–3.14
Missing	47	77		
Parity				
1	228 (77.0)	273 (47.1)	3.77	2.75–5.17
≥2	68 (23.0)	307 (52.9)	1.00	Ref
Gestational age (weeks)				
<34	13 (4.4)	2 (0.3)	18.9	4.23–84.6
34–<37	89 (30.1)	13 (2.2)	19.9	10.9–36.5
≥37	194 (65.5)	565 (97.4)	1.00	Ref
Smoking in early pregnancy				
Non smokers	240 (93.8)	490 (90.4)	1.00	Ref
1–9 cig/day	12 (4.7)	39 (7.2)	0.63	0.32–1.22
>9 cig/day	4 (1.6)	13 (2.4)	0.63	0.20–1.95
Missing	40	38		
Gender (boys)	153 (51.7)	299 (51.6)	1.01	0.76–1.33
Diabetes (yes)	6 (2.0)	2 (0.3)	5.98	1.20–29.8
Gestational diabetes (yes)	11 (3.7)	11 (1.9)	2.00	0.86–4.66
Involuntary childlessness for at least one year (yes)	25 (8.4)	36 (6.2)		0.82–2.37
Maternal country of origin Sweden	191 (71.0)	369 (65.8)		-
Other Nordic countries Western Europe, USA	10 (3.7)	20 (3.6)	-	
Australia, New Zeeland	3 (1.1)	6 (1.1)		
Former Eastern Europe	24 (8.9)			
Sub-Saharan Africa	6 (2.2)			
Middle East	27 (10.0)			
East Asia	5 (1.9)			
South America	3 (1.1)			
Missing	27			

^a^ no estimates were calculated.

**Table 2:** Spearman correlation coefficients (with *p*-values in parentheses) between the different PFAS compounds ^a^ in early pregnancy among 296 preeclampsia cases and 580 controls.

Cases	PFOA	PFOS	PFNA
PFOS	0.68 (<0.001)		
PFNA	0.28 (<0.001)	−0.06 (0.28)	
PFHxS	0.54 (<0.001)	0.57 (<0.001)	0.26 (<0.001)
Controls			
PFOS	0.68 (<0.001)		
PFNA	0.39 (<0.001)	0.14 (0.001)	
PFHxS	0.64 (<0.001)	0.63 (<0.001)	0.40 (<0.001)

^a^ PFOA: perfluorooctanoic acid; PFOS: perfluorooctane sulfonate; PFNA: perfluorononanoic acid; and PFHxS: perfluorohexane sulfonate.

**Table 3:** Serum concentrations of the different PFAS compounds (ng/mL) in early pregnancy among 296 preeclampsia cases and 580 controls divided by parity. For comparisons, only statistically significant (*p* < 0.05) are marked.

Exposure	Cases		Controls	
	Primiparous (*n* = 228)	Multiparous (*n* = 68)	Primiparous (*n* = 273)	Multiparous (*n* = 307)
	Mean/Median (min, max)	Mean/Median (min, max)	Mean/Median (min, max)	Mean/Median (min, max)
PFOA ^a,b^	3.10/2.82 ^c^ (0.55, 10.9)	2.28/1.96 ^c^ (0.42, 6.93)	3.01/2.83 ^d^ (0.39, 9.38)	2.08/1.81 ^d^ (0.40, 9.34)
PFOS ^a,b^	14.8/12.9 (2.15, 50.0)	14.3/10.9 (1.49, 66.6)	14.7/12.4 ^d^ (0.52, 54.5)	11.8/9.36 ^d^ (1.13, 47.0)
PFNA ^a,b^	0.55/0.47 (0.14, 2.82)	0.49/0.44 ^e^ (0.15, 1.27)	0.54/0.46 ^d^ (0.03, 3.51)	0.46/0.38 ^d,e^ (0.06, 3.30)
PFHxS ^a,b^	0.77/0.66 ^c^ (BDL ^b^, 9.41)	0.58/0.55 ^c^ (BDL ^b^, 2.32)	0.71/0.62 ^d^ (BDL ^b^, 4.48)	0.51/0.45 ^d^ (BDL ^b^, 1.93)

^a^ PFOA: perfluorooctanoic acid; PFOS: perfluorooctane sulfonate; PFNA: perfluorononanoic acid; and PFHxS: perfluorohexane sulfonate. ^b^ Detection limits (DL): PFOA: 0.04; PFOS: 0.12; PFNA: 0.03; PFHxS: 0.03. All values were above detection limits with the exception of PFHxS where three cases and six controls were below detection limits (BDL: Below detection limit). ^c^ Comparing primiparaous and multiparaous cases; PFOA *p* < 0.001 and PFHxS *p* = 0.01. ^d^ Comparing the primiparaous and multiparaous controls; all *p*-values < 0.001. ^e^ Comparing multiparaous cases and controls; PFNA *p* = 0.04.

**Table 4:** The associations between concentrations of PFAS ^a^ in maternal serum in early pregnancy and preeclampsia (PE). Odds ratios (OR) and 95% confidence intervals (CI) were obtained from logistic regressions.

PFAS ng/mL	Cases (*n*)	Controls (*n*)	Unadjusted		Adjusted ^b,c^	
			OR	95% CI	OR	95% CI
PFOA ^a^						
≤1.48	51	145	1.00	ref ^d^	1.00	ref
>1.48–2.12	52	145	1.02	0.65–1.60	0.94	0.56–1.57
>2.12–3.29	93	145	1.82	1.21–2.75	1.42	0.87–2.31
>3.29	100	145	1.96	1.30–2.95	1.13	0.68–1.87
PFOS ^a^						
≤6.66	67	145	1.00	ref	1.00	ref
>6.66–10.73	52	145	0.78	0.50–1.19	0.81	0.50–1.32
>10.73–18.09	95	145	1.42	0.96–2.09	1.23	0.78–1.93
>18.09	82	145	1.22	0.82–1.82	0.96	0.60–1.53
PFNA ^a^						
≤0.28	48	145	1.00	ref	1.00	ref
>0.28–0.40	72	145	1.50	0.97–2.31	1.34	0.81–2.22
>0.40–0.60	88	145	1.83	1.20–2.79	1.41	0.86–2.32
>0.60	88	145	1.83	1.20–2.79	1.51	0.91–2.50
PFHxS ^a^						
≤0.31	44	145	1.00	ref	1.00	ref
>0.31–0.53	67	145	1.52	0.98–2.38	1.37	0.82–2.28
>0.53–0.78	89	145	2.02	1.32–3.10	1.67	1.02–2.74
>0.78	96	145	2.18	1.43–3.34	1.38	0.83–2.30

^a^ PFOA: perfluorooctanoic acid; PFOS: perfluorooctane sulfonate; PFNA: perfluorononanoic acid; and PFHxS: perfluorohexane sulfonate. ^b^ Adjusted for maternal age (4 categories: ≤25, 26–<30, 31–<35, and >35 years), BMI in early pregnancy (4 categories: <20, 20–<25, 25–<30, ≥30 kg/m^2^), maternal smoking in early pregnancy (2 categories: yes and no), and parity (2 categories: 1 and ≥2). ^c^ Included 233 (79%) cases and 468 (81%) controls, i.e., those with complete data for all variables. ^d^ Reference category.
